# Efficient assembly of very short oligonucleotides using T4 DNA Ligase

**DOI:** 10.1186/1756-0500-3-291

**Published:** 2010-11-09

**Authors:** Daniel R Horspool, Robin JN Coope, Robert A Holt

**Affiliations:** 1 British Columbia Cancer Agency, Genome Sciences Centre, Suite 100, 570 West 7th Avenue, Vancouver, British Columbia V5Z 4S6, Canada; 2 Simon Fraser University, Department of Molecular Biology and Biochemistry, 8888 University Drive, Burnaby, British Columbia, V5A 1S6, Canada

## Abstract

**Background:**

In principle, a pre-constructed library of all possible short oligonucleotides could be used to construct many distinct gene sequences. In order to assess the feasibility of such an approach, we characterized T4 DNA Ligase activity on short oligonucleotide substrates and defined conditions suitable for assembly of a plurality of oligonucleotides.

**Findings:**

Ligation by T4 DNA Ligase was found to be dependent on the formation of a double stranded DNA duplex of at least five base pairs surrounding the site of ligation. However, ligations could be performed effectively with overhangs smaller than five base pairs and oligonucleotides as small as octamers, in the presence of a second, complementary oligonucleotide. We demonstrate the feasibility of simultaneous oligonucleotide phosphorylation and ligation and, as a proof of principle for DNA synthesis through the assembly of short oligonucleotides, we performed a hierarchical ligation procedure whereby octamers were combined to construct a target 128-bp segment of the beta-actin gene.

**Conclusions:**

Oligonucleotides as short as 8 nucleotides can be efficiently assembled using T4 DNA Ligase. Thus, the construction of synthetic genes, without the need for custom oligonucleotide synthesis, appears feasible.

## Findings

To address the high cost and limited efficiency of *de novo *gene synthesis we are exploring gene synthesis by ligation of deoxyribooligonucleotides (oligos), short enough to permit DNA assembly from a library of all possible oligos. This approach would serve to amortize oligonucleotide synthesis costs over many genes and potentially provide two methods of error reduction. First, by virtue of being short, the oligos themselves should be more accurate, and second, each ligation event requires a sequence match, which further reduces error. Short oligos are crucial to reducing the library size to a manageable set, however, ligation requirements make it unlikely that an ideal minimum could be used effectively. Efficient ligation of short oligos is essential for this approach, so we set out to investigate the optimal oligo lengths and conditions for which iterative ligations could be achieved using T4 DNA ligase.

It is known that both oligo length and sequence-dependent hybridization efficiency play a critical role in ligase efficiency and vary substantially between ligases [1,2]. The requirement for duplex DNA surrounding a nick to be sealed differs between the ligases. T7 DNA ligase can effectively join a hexamer and a nonamer on a complete template whereas Tth DNA Ligase is limited to an octamer and a nonamer on a complete template [3]. These variations suggest some ligases are better suited to joining short oligos. T4 DNA ligase was selected for the present study as it was known to ligate adjacent oligos as small as hexamers, hybridized for their complete length to a complementary template [4,5]. These previous results did not, however, examine the ligation of short, single stranded oligos in the absence of a completely hybridized template.

For the purposes of DNA synthesis, we were interested in the ligation of oligos to the end of a growing duplex such that the process could be iterated with a minimal overlap between oligos. In order to determine both the minimal oligo length and the minimum template overlap necessary for efficient ligation, we examined ligation of various labeled oligos onto a short overhang of immobilized double stranded DNA (dsDNA). We discovered that supplementary oligos greatly aided in the ligation of overhangs shorter than five nucleotides and demonstrated the benefit of this approach by assembling a 128-bp fragment of the human β-actin gene with octamers. The materials and methods used in the present study are as follows.

### Preparation of immobilized dsDNA

All oligos, including those 5'-biotinylated, 3'-FAM6, and 5'-phosphorylated were synthesized by Integrated DNA Technologies (IDT Inc., IA, USA). Immobilized double stranded DNA preparation involved purification of strepdavidin coated magnetic beads, binding of the biotinylated top strand, and then annealing of the complementary bottom strand. M-270 Streptavidin Dynabeads (Invitrogen) were washed three times with equal volume 2× B&W buffer (10 mM Tris-HCl pH 7.5, 1 mM EDTA, 2.0 M NaCl). DNA immobilization was performed by resuspension of the purified bead solution to 1× B&W buffer supplemented with 3.33 μM 5'-biotinylated oligo. After 20 minutes at room temperature with gentle rotation, two washes with equal volume of 1× B&W were performed to remove unbound oligo. Immobilized oligo was then hybridized to form dsDNA by resuspending the bead mixture in 10 mM Tris-HCl (pH 7.5), 0.1 M NaCl, 1 mM EDTA and 5 μM bottom strand oligo. Bead solutions were heated to 80°C for 5 minutes and cooled to room temperature. Final solutions were washed twice with equal volume TE 10:1 (pH 7.5) to remove excess bottom strand and quantified using a standard PicoGreen fluorescence assay (Molecular Probes, Invitrogen Detection Technologies).

### Ligation assays

25 μL ligations contained 0.2 μM immobilized dsDNA on beads, 0.002 to 20 μM 5'-phosphorylated, 3'-FAM oligo, 20 units of T4 DNA Ligase (NEB), 1× T4 DNA Ligase Buffer (50 mM Tris-HCl, 10 mM MgCl_2_, 1 mM ATP, 10 mM Dithiothreitol pH 7.5 @ 25°C). Ligations were carried out at 16°C for 1 hour and stopped by heat inactivation at 65°C for 10 minutes. Reactions at each oligo concentration were performed in triplicate. Control reactions with TE supplemented in lieu of ligase were also performed in triplicate at each oligo concentration measured. Washes with equal volume TE 10:1 (pH 7.5) were performed twice to remove excess unligated labeled-oligo. 20 μL of each reaction was quantified by 480 nm fluorescence measurements using a Wallac Victor Spectrophotometer (Perkin Elmer). Ligation rates at each concentration were corrected for background by subtraction of the average TE control fluorescence levels. Fluorescence units observed were converted to moles based upon the 480 nm fluorescence measurements of the pure labeled oligo at known molar concentrations.

### Ligation assays supplemented with a second olignucleotide

Ligations were performed using the same procedure as the single oligo reactions above with the addition of either 5 μL TE or 5 μL 20 μM of a second oligo and a modified reaction time. The final 30 μL reaction mixture contained 0.167 μM immobilized dsDNA on beads, 0.00167 μM to 16.7 μM 5'-phosphorylated 3'-FAM oligo, 3.33 μM second oligo, 20 units T4 DNA Ligase, and 1× T4 DNA Ligase Buffer (50 mM Tris-HCl, 10 mM MgCl_2_, 1 mM ATP, 10 mM Dithiothreitol pH 7.5).

### DNA synthesis using phosphorylated octamer precursors

Pooled ligation reactions consisted of 1.5 μM immobilized dsDNA on beads, 66.7 μM of each octamer, 1× T4 DNA ligase buffer (50 mM Tris-HCl, 10 mM MgCl_2_, 1 mM ATP, 10 mM Dithiothreitol, pH 7.5 @ 25°C) and 0.5 units/μL of T4 DNA Ligase. Reactions proceeded for four hours at 4°C and mixtures were then washed twice with equal volume of TE to remove unligated product and enzyme. In selected bead sets, this process was performed twice using the same conditions but with the octamers split into two groups to avoid a region of repeated sequence. BbsI digestion was performed by resuspending the bead solutions to 25 units BbsI (NEB), 50 mM NaCl, 10 mM Tris-HCl, 10 mM MgCl_2_, and 1 mM Dithiothreitol (pH 7.9 @ 25°C). Digestion was performed for three hours at 37°C followed by enzyme inactivation at 65°C for 20 minutes. Released DNA fragments were isolated by immediate aspiration from the hot digest mixture while a magnet was applied. The extracted mixture was cooled to 4°C for 5 minutes and the full volume was used in subsequent ligations. Pairwise ligation steps were performed by resuspending an immobilized bead solution with an adjacent digested fragment solution. Ligation reactions consisted of 1.5 μM immobilized DNA, the released DNA fragment (unknown concentration), 1× T4 DNA ligase buffer (50 mM Tris-HCl, 10 mM MgCl_2_, 1 mM ATP, 10 mM Dithiothreitol, pH 7.5 @ 25°C) and 0.5 units/μL of T4 DNA Ligase. Reactions proceeded for four hours at 4°C and mixtures were then washed twice with equal volume of TE to remove unligated product and enzyme. Digest and ligation steps were repeated as necessary to complete the pair-wise construction process.

### DNA synthesis using non-phosphorylated octamer precursors

Simultaneous phosphorylation-ligation reactions consisted of 1.5 μM immobilized dsDNA on beads, 22.5 μM of each octamer, 1× T4 DNA ligase buffer (50 mM Tris-HCl, 10 mM MgCl_2_, 1 mM ATP, 10 mM Dithiothreitol, pH 7.5 @ 25°C), 0.5 units/μL of T4 Polynucleotide Kinase (NEB) and 0.5 units/μL of T4 DNA Ligase. Reactions proceeded for 2 hours at 24°C, followed by two washes with equal volumes of TE. BbsI digestion was performed as described above with no modifications.

### DNA sequencing

2 μL of the final bead solution was amplified using Platinum PCR SuperMix (Invitrogen) according to manufacture's directions. The forward primer (5'-GCAGTTCCGGATCCATCTAGA-3') and reverse primer (5'-TGCCAGATTTTCTCCATGTCGT-3') were designed to match a segment of the bead adaptor and the end of the target construct, respectively. PCR amplification was performed on a DNA Engine Dyad 2 Peltier Thermal Cycler (BIO-RAD) with parameters of 25 cycles at 96°C for 10 seconds (with an additional 2 minutes for the first cycle), 53°C for 5 seconds and 60°C for 1 minute, followed by incubation at 4°C. PCR products were analyzed by PAGE (20% 19:1 Acryl-Bis, 1× TAE, 0.1% APS) and visualized by SYBRGreen staining. PAGE purified PCR products were cloned into a Zero-Blunt TOPO kit vector (Invitrogen) and capillary sequenced on a 3730 × l DNA Analyzer (Applied Biosystems) using standard methods. All successful sequence reads were analyzed using the sequence-alignment tool ClustalX, and seqeuences were confirmed by direct inspection of electropherograms.

The results obtained in the present study are as follows.

### Ligation of a dsDNA with an overhang to a complementary ssDNA fragment requires a minimum five base pair duplex

**Figure 1:**
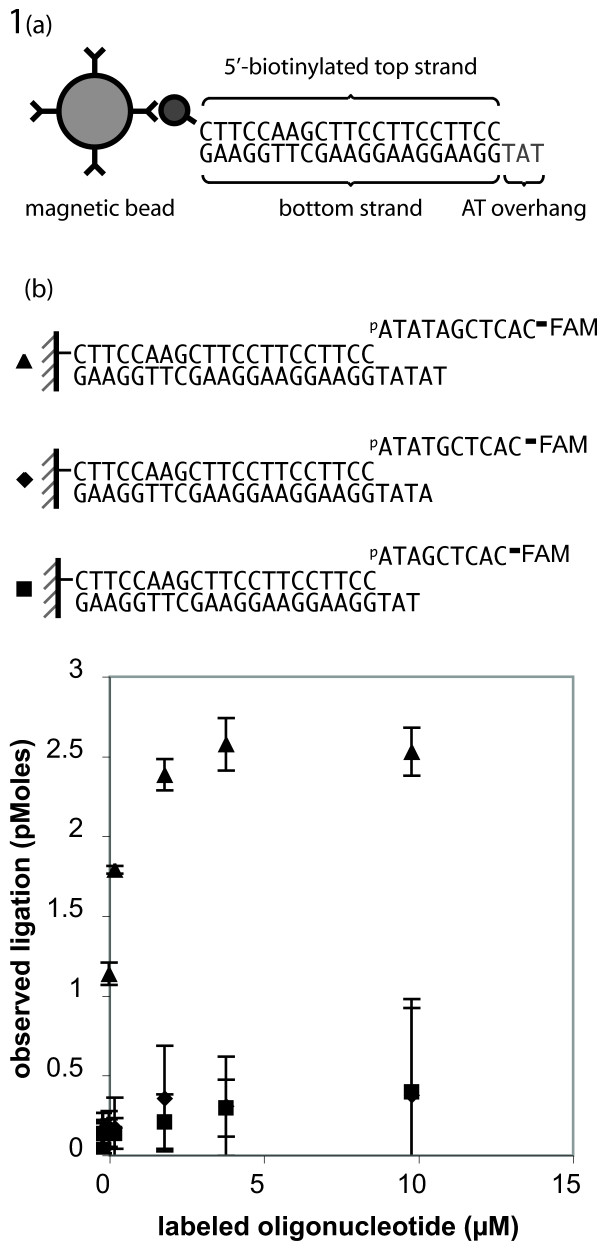
**Evaluation of minimal oligonucleotide substrate requirements for T4 DNA ligase.** (a) Schematic diagram of an immobilized DNA strand used in ligation assays and DNA construction. M-270 Dynabeads (Invitrogen) are attached through a streptavidin-biotin linkage to the 5' end of a double stranded DNA. The free end is designed with a variable 5' overhang, complementary to labeled oligonucleotides used in ligation. An additional BbsI restriction site and a forward primer site are included in the case of DNA construction. **(b) **Increasing concentrations of 5'-phosphorylated, 3'-fluorescently labeled oligonucleotide are ligated to 5 pmoles of immobilized dsDNA with a complementary overhang. Reactions were performed for one hour at 16°C and washed with TE to remove unligated substrate. Successful ligation kinetics are observed at the 5-bp duplex length (▲), but no significant ligation occurs at lengths of 4-bp (■) or 3-bp (◆).

We investigated the addition of a labeled oligo which, when annealed to its dsDNA counterpart, produced a double-stranded region of 3- to 5-bp (Figure 1a). Ligation for one hour at 16°C showed standard saturation kinetics on overhangs of 5-nt or more, however, reactions with overhangs of 4-nt or less showed no detectable ligation (Figure 1b). Additional experiments with temperature variations of 4, 8, and 12°C and a time extension to 16 hours showed relatively little improvement in ligation efficiency of these short fragments. These data show that T4 DNA ligase has an essential requirement for a DNA duplex, of at least five base pairs surrounding the nick, for efficient sealing.

### Efficiency of ligations of dsDNA-oligonucleotides with duplexes shorter than five base pairs can be improved by the addition of a supplementary oligonucleotide

**Figure 2:**
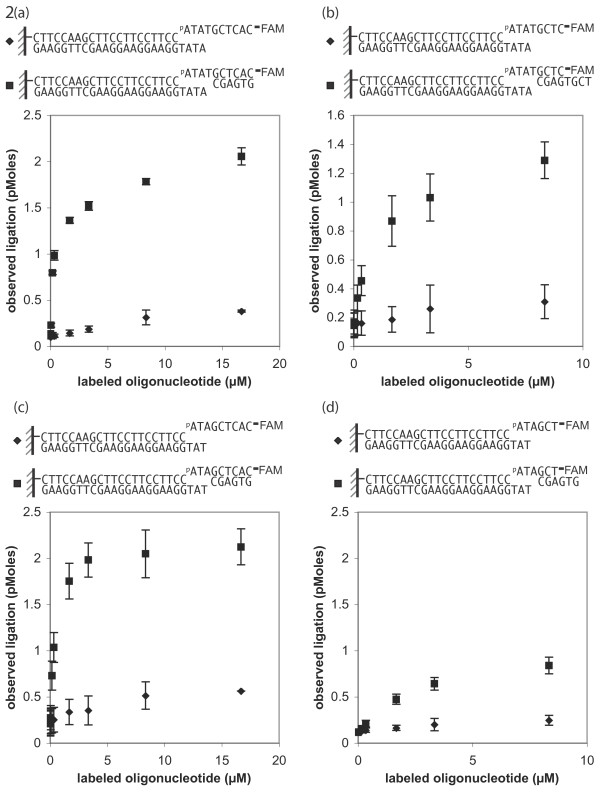
**Enhancement of T4 DNA ligase activity by supplemental oligonucleotides.** (a) Unsuccessful 4-bp duplex reactions could be salvaged by utilizing a supplementary oligonucleotide, designed to complement the first oligonucleotide-dsDNA duplex but is unphosphorylated to prevent ligation of itself. Two hour ligation of the 4-bp reaction at 16°C supplemented with 3.33 μM of the hexamer, shows successful ligation (■) while reactions without the supplementary hexamer show no activity (◆). **(b) **Ligation reaction of an octamer supplemented with a second octamer in which one is used for ligation and the other is used to extend the duplex. A two hour ligation at 16°C of serial concentrations of the octamer with 3.33 μM of the supplementary octamer shows significant ligation (■) compared to reactions without the supplemental octamer (◆). **(c) **Unsuccessful 3-bp duplex reactions could be salvaged by utilizing a supplementary hexamer that hybridized at all six positions. A two hour ligation of the 3-bp reaction at 16°C with 3.33 μM supplementary hexamer shows successful ligation (■) while reactions without the supplementary hexamer show no activity (◆). **(d) **Ligation using a hexamer pair at 4°C for 16 hours shows limited improvement (■) compared to the unsupplemented (◆) control.

To see if the short duplex reaction could be improved, the above reactions were repeated with the addition of a second, supplemental oligo, complementary to the linker of the oligo to be ligated. It was expected that correct hybridization of the three components (the dsDNA substrate, the 5'-phosphorylated 3'-FAM-6 oligo, and the second supplemental oligo, complementary to the labeled oligo) would result in successful ligation of the labeled oligo. In this manner, ligation efficiency for a 4-nt overhang reaction was markedly enhanced (Figure 2a). Shorter oligos were then tested to determine the lower limit of oligo length for successful ligation. A pair of octamers, producing a 4-bp duplex with 4-nt 5' overhangs on both sides, displayed successful ligation (Figure 2b). Such an arrangement in DNA construction would allow for the iterative assembly of octamers by successive 4-bp increments.

With the improvement of the 4-nt overhang reaction and the success of octamers, a further reduction to 3-nt was examined. We found that a 3-nt overhang reaction could be enhanced with the addition of a supplemental hexamer if that hexamer hybridized at all six positions (Figure 2c). A pair of hexamers was then examined such that when hybridized, the hexamer pair produced a 3-bp duplex with 3-nt overhangs on both sides. Such an arrangement would allow for the iterative assembly of DNA in 3-bp increments. Unfortunately, no significant ligation was observed. Intensifying the ligation conditions to 4°C and greater than 16 hours incubation time provided only a very modest improvement (Figure 2d). It has been shown that hexamer ligations are possible, but results are inconsistent and reactions must be performed under more facilitating conditions [4]. We suspect that the lack of efficient ligation is a collective consequence of there being a requirement for three different strands to anneal together long enough for T4 DNA Ligase to act, a reduced Tm of the shorter annealing sequences, and reduced contacts between T4 DNA Ligase and its substrates. We cannot exclude the possibility that the fluorescently labeled end of the hexamer may interfere with oligo entry into the catalytic pocket of T4 DNA Ligase.

### Construction of a 128 mer using four sets of octamers

**Figure 3:**
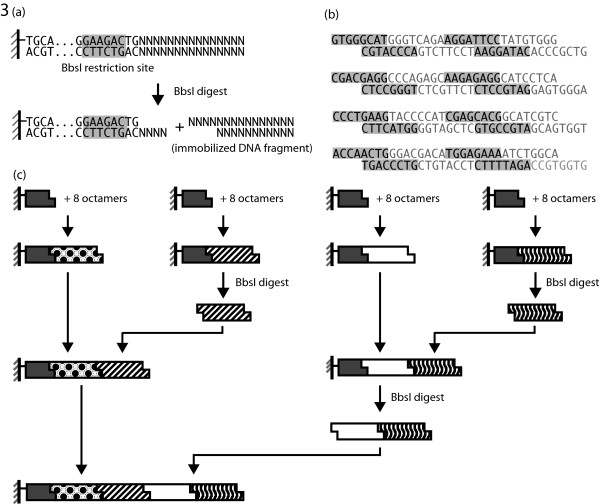
**Strategy for DNA synthesis by ligation of octamers.** (a) Experimental design a DNA adaptor containing a BbsI restriction site at the ligation end. An immobilized product can be purified through washing and released through BbsI digestion while maintaining a 4-nt overhang allowing it to be used in further ligation reactions. **(b) **Mapping of target construct into four 32-bp fragments. Each fragment is made of eight octamers and has ends complementary to the next fragment in the series. **(c) **Schematic overview of the pair-wise construction process. Four groups of octamers are ligated in pooled reactions to produce 32-bp intermediates as outlined in (b). Selected intermediates are then mobilized and ligated to neighboring fragments. A second round repeats the process to produce one fragment containing all four intermediates.

From the ligation experiments, it was concluded that DNA synthesis was feasible with octamers. To improve the pace of such a method, a hierarchical approach was designed in which multiple intermediate fragments could be constructed from octamers in parallel and then combined in a repeated pair-wise manner. The solid-support used to anchor growing intermediate fragments was designed such that digestion with BbsI would release any attached fragment while retaining a 4-nt overhang (Figure 3a). Released intermediates could then be used in further ligations. Four distinct bead sets were created each with a unique 4-nt overhang (Figure 3b). The overhangs for the solid support adaptors were constructed to be complementary to evenly distributed regions of the 128-bp target such that eight octamers, overlapping in 4-nt frame shifts, would tile between each region.

**Figure 4:**
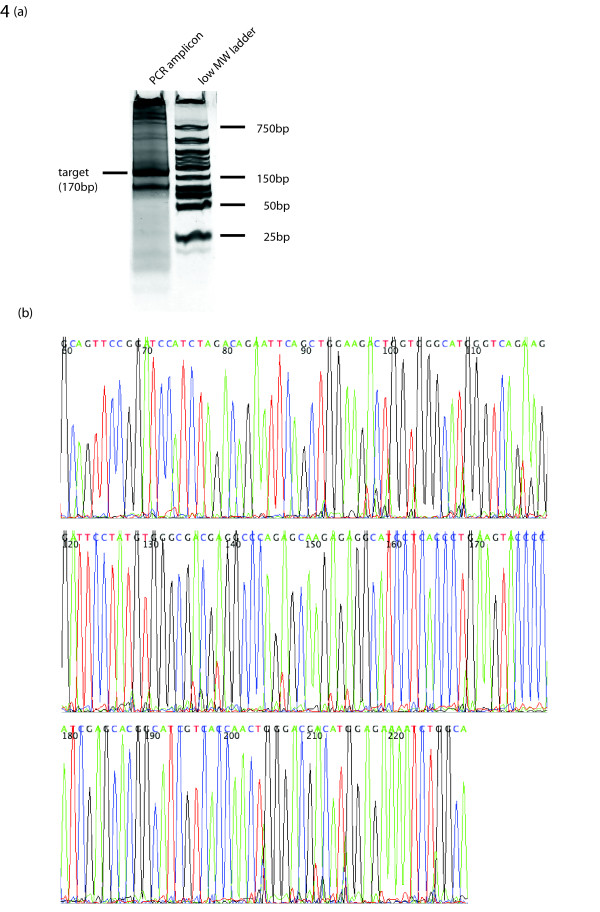
Validation of DNA synthesis by ligation of octamers. (a) PCR amplification of construct using 2 μL of the immobilized DNA product. The forward primer was identical to a region in the adaptor DNA attached to the first bead and the reverse was complementary to the tail end of the final intermediate to be ligated. **(b) **Electropherogram of a successfully cloned and sequenced product. The first 42-bp represent those of the adaptor while the latter 128-bp are those of the target fragment assembled with octamers.

In the first step, pooled ligation reactions were performed with the solid support and nine octamers. We suspected from our T4 DNA Ligase experiments that each octamer would act as a partial template for the next with the exception of the ninth octamer which would merely serve to allow the last octamer in the set of eight to ligate. To avoid problematic regions of non-unique complementary ends found in the octamer pools, two of the pooled ligations (C and D) were performed in two steps, avoiding the repeated region. Each of the four products from this process, A, B, C, and D, were expected to be 32-bp. In the second phase of construction, fragments B and D were detached from their solid support using BbsI and then ligated to the immobilized fragments, A and C, to produce fragments AB and CD. A third digest and ligation phase, identical to the second, released the fragment CD and ligation with the immobilized AB intermediate produced the product ABCD (Figure 3c). PCR amplification of the final product was then performed directly from the immobilized dsDNA (Figure 4a). Sequencing of the 170-bp target band verified a single product containing the 42-bp adaptor and 128-bp construct (Figure 4b). Sequencing of the second, smaller band revealed a product missing one of the 32-bp intermediate fragments.

### Simultaneous phosphorylation and ligation

**Figure 5:**
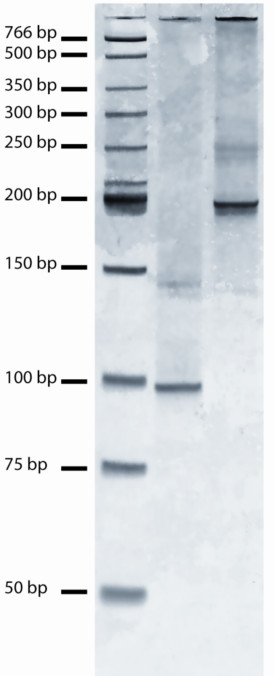
**PCR amplification of DNA segments constructed by simultaneous oligo phosphorylation/ligation**. Shown are size markers (NEB LMW ladder, lane 1), a 92 bp segment constructed by simultaneous phosphorylation/ligation of precursor octamers (lane 2), which was then joined to a second 92 bp segment constructed by the same approach, to yield a 188 bp product (lane 3).

Cost is an important consideration in gene synthesis applications. While we have not yet explored cost restriction by minimizing reaction volumes, we have shown that it is possible to undertake simultaneous oligo phosphorylation and ligation, which eliminates the need requirement of expensive pre-phosphorylated oligos. Using the same solid support method described above, two 92-nt segments were constructed, each by three consecutive rounds of phosphorylation/ligation of pooled octamers. This was followed by a final BbsI digest to release the segments from their solid supports and a final ligation to join the two segments via their complementary 4-nt ends. PCR amplication of the first segement alone, plus the first and second segments joined together, produced products of expected size (Figure 5).

To conclude, *de novo *gene synthesis offers the ability to optimize genes for unnatural hosts, alter existing or append new restriction sites, create chimeric fusion proteins, or even produce genes for completely artificial transcripts. This approach can be more powerful and practical than conventional genetic manipulation methods [6], but its use has been limited by the high cost associated with producing error free DNA constructs from expensive and error prone oligo components [7-9]. In principle, a library of all possible oligos could provide a reusable stock for synthesis of many different longer DNA sequences. The present study illustrates that this approach is, in principle, possible, usingT4 DNA Ligase to combine small oligos with short overhangs.

Many DNA ligases have been isolated and characterized. While their functions are similar, their structure and cofactor dependencies vary dramatically [10] and there is dissimilar fidelity and efficiency among ligases [2]. Specifically, T4 DNA Ligase has been known for some time to be capable of joining oligos as small as pentamers and hexamers on a complete template [5], however ligases such as Tth DNA Ligase are severely inefficient at the hexamer level [3]. Our experiments suggested that T4 DNA Ligase could not efficiently catalyze the joining of an oligo hexamer to a dsDNA with a complementary overhang of three nucleotides. Binding experiments with fluorescently labeled oligos confirmed this limitation. While ligation of hexamers to 3-nt overhangs is possible with the addition of a second complementary hexamer, its low efficiency and extensive reaction time suggests DNA construction using hexamers would be extremely inefficient. We show that octamers arranged in 4-bp overlapping frames are the next most viable alternative. Although the length of an octamer increases the size of the oligo library sixteen fold (to 65,536) octamers provide the best potential for successive ligations while still being short enough to consider the preconstruction of an oligo library. A reduced set of octamers that represent only selected codon combinations may yield a useful and practical library.

We suspect the accuracy of the 128-bp fragment we constructed, in the absence of any error correction, is due to the accuracy in synthesizing of short oligos and the ability of T4 DNA ligase to discriminate any base-pair mismatches that may exist. It is known that mismatches significantly reduce ligation efficiency [3,11]. We tested pools of up to nine unpurified octamers and showed that these could be correctly organized and combined by T4 DNA Ligase without incorporating errors into the final product.

Despite the benefits of this new approach, there are obstacles that need to be considered during selection of a set of precursor oligos. Octamers that are complete palindromes will self-hybridize, and those with 4-nt palindromic ends will result in self-polymerization. Further, repetitive sequences longer than 8 bp cannot be synthesized from octamers, and cleavage from the solid support by BbsI digestions requires that the recognition site for this enzyme not be encoded elsewhere within the assembled sequence Thus, design is best undertaken using an algorithimic approach, to group octamers into subsets that are assembled hierarchically. By this approach problematic sequences can often be mitigated by placing them at the junction between synthesized sub-segments. In principle, it is also possible to include in the design longer, custom oligos that span problematic sequences, although at increased cost.

This present study demonstrates the feasibility of DNA synthesis by assembly of short oligo precursors. Future work will involve improving the efficiency, perhaps by adapting these methods to microfluidic devices.

## Competing interests

The authors declare that they have no competing interests.

## Authors' contributions

DRH generated and analyzed the data presented here. DRH, RJNC and RAH conceived of the study and co-wrote the manuscript. RAH directed the research.
